# Within-farm dynamics of ESBL-producing *Escherichia coli* in dairy cattle: Resistance profiles and molecular characterization by long-read whole-genome sequencing

**DOI:** 10.3389/fmicb.2022.936843

**Published:** 2022-07-28

**Authors:** Maitane Tello, Medelin Ocejo, Beatriz Oporto, José Luis Lavín, Ana Hurtado

**Affiliations:** ^1^Department of Animal Health, NEIKER – Basque Institute for Agricultural Research and Development, Basque Research and Technology Alliance (BRTA), Derio, Bizkaia, Spain; ^2^Department of Applied Mathematics, NEIKER – Basque Institute for Agricultural Research and Development, Bioinformatics Unit, Basque Research and Technology Alliance (BRTA), Derio, Bizkaia, Spain

**Keywords:** antimicrobial resistance, dairy cattle, extended-spectrum β-lactamases, ESBL-producing *Escherichia coli*, minimum inhibitory concentration, whole-genome sequencing, long-read WGS

## Abstract

A longitudinal study was designed in five dairy cattle farms to assess the within-farm dynamics of ESBL-/AmpC-/carbapenemase-producing *E. coli* and their resistance profiles, along with the genes conferring the resistance phenotypes. Twelve samplings were performed over a period of 16 months, collecting rectal feces from apparently healthy animals in three age groups (calves, heifers, and lactating cows) that were subjected to selective isolation in cefotaxime-containing media. Minimum inhibitory concentrations were determined by broth microdilution for 197 cefotaxime-resistant *E. coli* (1–3 isolates per age group and sampling date), and 41 of them were selected for long-read whole-genome sequencing. Cefotaxime-resistant *E. coli* were detected in the five farms, but isolation frequency and resistance profiles varied among farms and age groups. The genetic profiling of a selection of isolates recovered in two of the farms was described in full detail, showing the predominance of a few genomic subtypes of *E. coli* in one farm (F1) and great variability of strains in another one (F4). Two predominant distinct strains carrying the *bla*_CTX-M-1_ gene in IncX1 plasmids successively spread and persisted in F1 over a prolonged period. In F4, 13 different MLST types carrying a high diversity of ESBL-encoding genes in 6 different plasmid types were observed, probably as the result of multiple source contamination events. In both farms, the presence of certain plasmid types with the same repertoire of ARGs in different *E. coli* STs strongly suggested the occurrence of horizontal transfer of such plasmids among strains circulating within the farms. Considering the public health importance of ESBL-producing *E. coli* both as pathogens and as vectors for resistance mechanisms, the presence of β-lactamase- and other AMR-encoding genes in plasmids that can be readily transferred between bacteria is a concern that highlights the need for One Health surveillance.

## Introduction

Cephalosporins (third- and higher-generation) and carbapenems are critically important antimicrobials for human medicine since in some instances, they are either the sole or one of the limited therapies available to treat multidrug-resistant (MDR) bacteria in human infections (WHO, 2019). *E. coli* strains can become resistant to these antimicrobials by the acquisition of antimicrobial resistance genes (ARGs) coding for enzymes like extended-spectrum β-lactamases (ESBL), AmpC cephalosporinases, and carbapenemases (CP). ESBL and AmpC enzymes are capable of hydrolyzing various β-lactam antibiotics such as penicillins, third- and higher-generation cephalosporins, and monobactams, while AmpC enzymes are additionally active against cephamycins and resistant to inhibition by clavulanate. CPs confer resistance to a broad spectrum of β-lactams, including carbapenems, a last resort for treating MDR Gram-negative bacterial infections.

ESBL/AmpC-producing *E. coli* are widely distributed in livestock (Poirel et al., 2018; Dantas Palmeira and Ferreira, 2020), but their contribution as a source of human infection remains controversial (Collis et al., 2019). On the other hand, CP-producing *E. coli* are still scarcely detected in cattle (Madec et al., 2017; Kock et al., 2018; EFSA and ECDC, 2021). The spread of ESBL-/AmpC-/CP-producing *E. coli* can be the result of the selection of resistance (usually at the intestinal level) under the pressure of antibiotic usage, and the dissemination of such resistant bacteria by cross-contamination of fecal material among animals (Seiffert et al., 2013). In a previous cross-sectional survey conducted in the Basque Country in 2014–2016 to study the herd-level prevalence of ESBL-/AmpC- and carbapenemase-producing commensal *E. coli* in ruminants, a higher prevalence was detected in dairy cattle compared with beef cattle and sheep (Tello et al., 2020). However, the association of animal age with the likelihood of ESBL-/AmpC-producing *E. coli* shedding was not investigated. Besides, cross-sectional studies do not provide information on the long-term dynamics of bacterial shedding, which is relevant for understanding their potential for spread and persistence within the farm. Longitudinal data on fecal shedding of ESBL-/AmpC-producing *E. coli* within farm animals remains limited. Other longitudinal studies performed on dairy cattle either focused on a single farm, were short time-framed, or applied different approaches and methodologies (Hordijk et al., 2013, 2019; Horton et al., 2016; Gay et al., 2019; Plassard et al., 2021), but none combined long-term monitoring with a detailed genomic analysis.

To further explore the epidemiology of ESBL-/AmpC-producing *E. coli* on dairy cattle farms, we studied the dynamics of fecal shedding in animals from different age groups in five dairy cattle farms in the Basque Country. To increase detection efficiency, selective pre-enrichment was used. Phenotypic antimicrobial susceptibility of isolates recovered from the five farms was tested, and in-depth genome characterization of isolates from two of the farms was performed using long-read sequencing (Oxford Nanopore Technologies, ONT) to investigate ARG transmission dynamics. Bacterial chromosomes and plasmids were reconstructed and typed.

## Materials and methods

### Study design

A longitudinal study was carried out in dairy cattle farms in the Basque Country (northern Spain) to monitor the occurrence of ESBL-/AmpC-/CP-producing *E. coli* in apparently healthy animals. Five commercial farms (designated F1, F2, F3, F4, and F5), representative of the style of farming in the region, were selected to be enrolled in the study. Farms were located in the three counties of the Basque Country, and the distance between farms ranged from 15–25 km for those located within the same county (i.e., F3-F4 and F1-F2, respectively) and up to 160 km (F4-F5). Before the study started, our team paid a visit to each farm and, in the presence of the farm veterinary clinicians, farmers were interviewed face to face using a questionnaire that addressed general information about farm characteristics, management practices, vaccine programs, and antimicrobial drug use. Farm size based on the combined number of lactating and dry cows, heifers, and calves, ranged between 140 and 320 animals (mean = 240), with the number of lactating cows ranging from 75 (F5) to 200 cows (F1).

Monthly visits over a 1 year-period were planned for fecal sample collection. However, one of the farms (F5) dropped out after five samplings due to operational changes; samplings in the other four farms were interrupted midway through the study due to the COVID-19 pandemic and resumed at different times after the lockdown to complete the 12 samplings scheduled. Overall, the collection of fecal samples commenced in February 2019 and ended in October 2020, and extended over 16–17 months within individual farms. Samples were collected from apparently healthy animals from different age groups defined according to the different management practices, i.e., 1–5 month-old calves, 5–22 month-old heifers, and lactating cows. At each sampling time, rectal fecal samples (minimum of 5 g) were collected with a gloved hand from five animals randomly selected within each age group, and analyzed in a single 25 g pool per age group (5 g per animal). In seven time points, heifers could not be sampled in the two farms (five sampling times in F2 and two in F4) that raised heifer replacements at a breeding center. A total of 760 rectal fecal samples were collected and analyzed in 152 pools. Additionally, environmental slurry samples were also collected from F3 and F4 (two samplings each).

### Selective isolation of ESBL-/AmpC- and carbapenemase (CP)-producing *Escherichia coli*

Upon arrival, samples were refrigerated at 4°C and sample processing was carried out within 3 days after collection, at the latest. Pooled fecal samples (25 g) were thoroughly mixed, diluted 1:10 in buffered peptone water (BPW, bioMérieux), and incubated at 37°C for 20 ± 2 h. For the isolation of ESBL-/AmpC-producing *E. coli*, two loops (20 μl) of BPW were subcultured onto MacConkey agar supplemented with 1 mg/l of cefotaxime and incubated at 37°C for 20 ± 2 h. Two morphologically different colonies per plate were harvested and confirmed as *E. coli* by species-specific real-time PCR detection of the *uidA* gene (Frahm and Obst, 2003).

For the isolation of CP-producing *E. coli*, two loops (20 μl) of BPW were subcultured onto MacConkey agar without antibiotics. A loopful of grown colonies was then harvested for DNA extraction and subjected to a real-time PCR amplification screening targeting the CP-coding genes *bla*_NDM_, *bla*_VIM_, *bla*_KPC_, and *bla*_OXA-48_ (Ellington et al., 2016). If any of these genes tested positive, a loopful of bacterial growth from the MacConkey agar was subcultured on ChromID^®^ Carba Smart selective agar plates (bioMérieux), and isolated colonies were identified by *uidA* gene detection as above.

### Antimicrobial susceptibility testing by broth microdilution

Between 1 and 3 isolates per plate were selected and tested to assess antimicrobial susceptibility. Minimum inhibitory concentrations (MICs) were determined by broth microdilution using two Sensititre^®^ MIC susceptibility plates (EUVSEC1 and EUVSEC2, Thermo Fisher Scientific) following the recommendations in Commission Implementing Decision 2013/652/EU^1^ concerning antimicrobials and dilution ranges, and the results were interpreted using epidemiological cutoff values (ECOFF). For antimicrobials with no ECOFFs assigned at the time, the results were interpreted as follows: for temocillin, ECOFF was fixed at 16 mg/l based on 2020/1729/EU; for azithromycin, 16 mg/l was used as a reference based on the bibliography (Sjölund-Karlsson et al., 2011; Clinical and Laboratory Standards Institute, 2015).

### Whole-genome sequencing and bioinformatic analyses

Based on their phenotypic AMR profile, sampling time, and age group isolation source, 41 isolates (27 from F4, 11 from F1, and one each from F2, F3, and F5) were selected for WGS. For in-depth genome characterization, genomic DNA was extracted from pure cultures using NZY Microbial gDNA Isolation kit (NZYtech) and subjected to long-reads (Oxford Nanopore Technologies, ONT) WGS. For ONT sequencing, a library was prepared using the Ligation Sequencing Kit (SQK-LSK109). Native barcoding genomic DNA kits (EXP-NBD104 and EXP-NBD114) were used for sample multiplexing except for three isolates that were sequenced in singleplex. Libraries were run in FLO-MIN106 (R9.4.1) or FLO-MIN111 (R10.3) flow cells on a MinION Mk1C device (ONT). For validation purposes, five isolates also underwent short-reads (Illumina) WGS; genomic DNA was submitted to Eurofins Genomics, where libraries were prepared based on the NEBNext Ultra II FS DNA library prep kit (Illumina) and sequenced with Illumina NovaSeq 6,000 (150-bp paired-end reads). The output files generated by ONT sequencing were basecalled in high-accuracy mode (HAC) and quality-filtered using Guppy (Qscore >7 in v4.2 and v4.3, and Qscore >8 in v5.0). Then, reads were adapter-trimmed and filtered by length and quality, as described before (Tello et al., 2022) and the resulting fastq reads were *de novo* assembled using Unicycler (Wick et al., 2017b). For one particular sample, Flye assembler (Kolmogorov et al., 2019) was used after retrieving inconsistent results in the draft genome generated with Unicycler, and the resultant assembly was the one further used in this study. For isolates sequenced by both technologies, Illumina reads were pre-processed for assembly as described elsewhere (Tello et al., 2020) and the outputs were further used to generate hybrid Nanopore-Illumina assemblies with Unicycler (Wick et al., 2017b). As previously described (Tello et al., 2022), isolates were subjected to *in silico* typing to determine their serogroup and phylogroup. MLST profiles were determined from unassembled long-reads using Krocus (Page and Keane, 2018). New sequence type (ST) assignations were obtained after submitting WGS reads to the Enterobase database (Zhou et al., 2020). Draft genomes were processed to predict plasmid- and chromosome-derived contigs using PlasFlow (v.1.1; Krawczyk et al., 2018). Molecular characterization of the isolates, including screening of ARGs, chromosomal point mutations associated with AMR, virulence factors detection, and plasmid replicon identification were performed, as previously described (Tello et al., 2020). Databases used for molecular characterization (ResFinder, PointFinder, PlasmidFinder, and ecoli_vf) were all updated on 20 October 2021. ResFinder hits were filtered at 90% coverage and identity and those with values below 100% were individually revised for frameshifts and amino acid changes, removing those considered not potentially functional. Virulence genes were filtered at 75% identity and 95% coverage, and the pattern of presence/absence of these genes was used as a typing scheme for genetic diversity. Genome annotations were carried out with Prokka (Seemann, 2014) and RAST (Aziz et al., 2008), and were graphically represented using SnapGene v.5.2.4.^2^ Genome alignments were performed using MAUVE (Darling et al., 2010) in Geneious Prime v. 2020.2.4 software.^3^ Blast Ring Image Generator (BRIG) v.0.95 was used for plasmid structural comparison (Alikhan et al., 2011).

Phenotypic resistance profiles and the genetic determinants of resistance (GDR) in each sequenced sample (chromosome and plasmids) were represented in heatmaps. The plasmid heatmap was graphed along with a dendrogram illustrating the similarity among plasmids based on their AMR pattern. The hierarchical clustering analysis for the dendrogram was performed with the unweighted pair-group method with arithmetic mean (UPGMA) based on the Jaccard distance matrix, using the function hclust (v.3.6.1) of the R statistical package v.3.6.3. To identify the shared and unique phenotypic antimicrobial resistance profiles among the different age groups within each farm, Venn diagrams were constructed with the online tool InteractiVenn (Heberle et al., 2015).

### Statistical analyses

To evaluate differences between age groups and farms in the shedding prevalence of cefotaxime-resistant *E. coli* and in the occurrence of phenotypic antimicrobial resistance for each antimicrobial, multivariate logistic regressions were performed including age group and farms as the explanatory variables. Adjusted odds ratios (OR_adj_) were used as the measure of association between positivity and the explanatory variables and were expressed with their confidence interval at 95% (95% CI). Differences were considered statistically significant if *p* < 0.05. Simpson indices were estimated to calculate the diversity of phenotypic antimicrobial resistance profiles for each farm.

## Results

### Farms’ descriptive data derived from the questionnaire

Following common practice in dairy farms in the Basque Country, all farms were closed production systems where replacement heifers originated from the same farm. Two of the farms (F2 and F4) raised their heifer replacements off-site in two different breeding centers. In both cases, animals leave the farm at 3–4 months of age and return already pregnant a few months before calving. A blanket antimicrobial treatment program was routinely used at dry-off that included the intramammary application of antimicrobials and teat sealant. The antimicrobials used for intramammary dry-cow therapy (DCT) were benzylpenicillin-benethamine/framycetin sulfate (Mamyzin) in F1 and F2, and cephapirin benzathine (Cefa-safe) in F3, F4, and F5. Farms participating in the study also used antimicrobials belonging to 12 antimicrobial drug classes for the treatment of disease in calves and cows. The antimicrobials most commonly used were third- and fourth-generation cephalosporins, followed by fluoroquinolones, and tetracyclines. Other antimicrobials used included penicillins, aminoglycosides, macrolides, and sulfonamides. Parenteral administration of fluoroquinolones was the most common treatment for mastitis during lactation in all except farm F2 where mastitis was not treated with antimicrobials. Third- and fourth-generation cephalosporins were the most common drugs used to treat reproductive diseases, diarrhea, and lameness that warranted systemic antimicrobial treatment. Reproductive diseases for which the producer opted to use antimicrobials included metritis, retained placenta, or other diseases related to reproduction.

Vaccination programs were quite different among farms. For example, vaccination against mastitis was only performed in F1. The vaccination program in F1 included vaccines against Infectious Bovine Rhinotracheitis (IBR), clostridia, and mastitis; IBR, *Leptospira,* and respiratory pathogens (Parainfluenza, bovine respiratory syncytial virus - BRS, *Mannheimia*) in F2; no vaccines at all were used in F3; IBR, Bovine viral diarrhea virus, and clostridia in F4; and clostridia, diarrhea in calves, and respiratory pathogens (Parainfluenza, BRS, *Mannheimia*) in F5.

### Cefotaxime-resistant *Escherichia coli* isolates were frequently recovered in the five dairy cattle farms, but differences were found among age groups and farms

**Figure 1:**
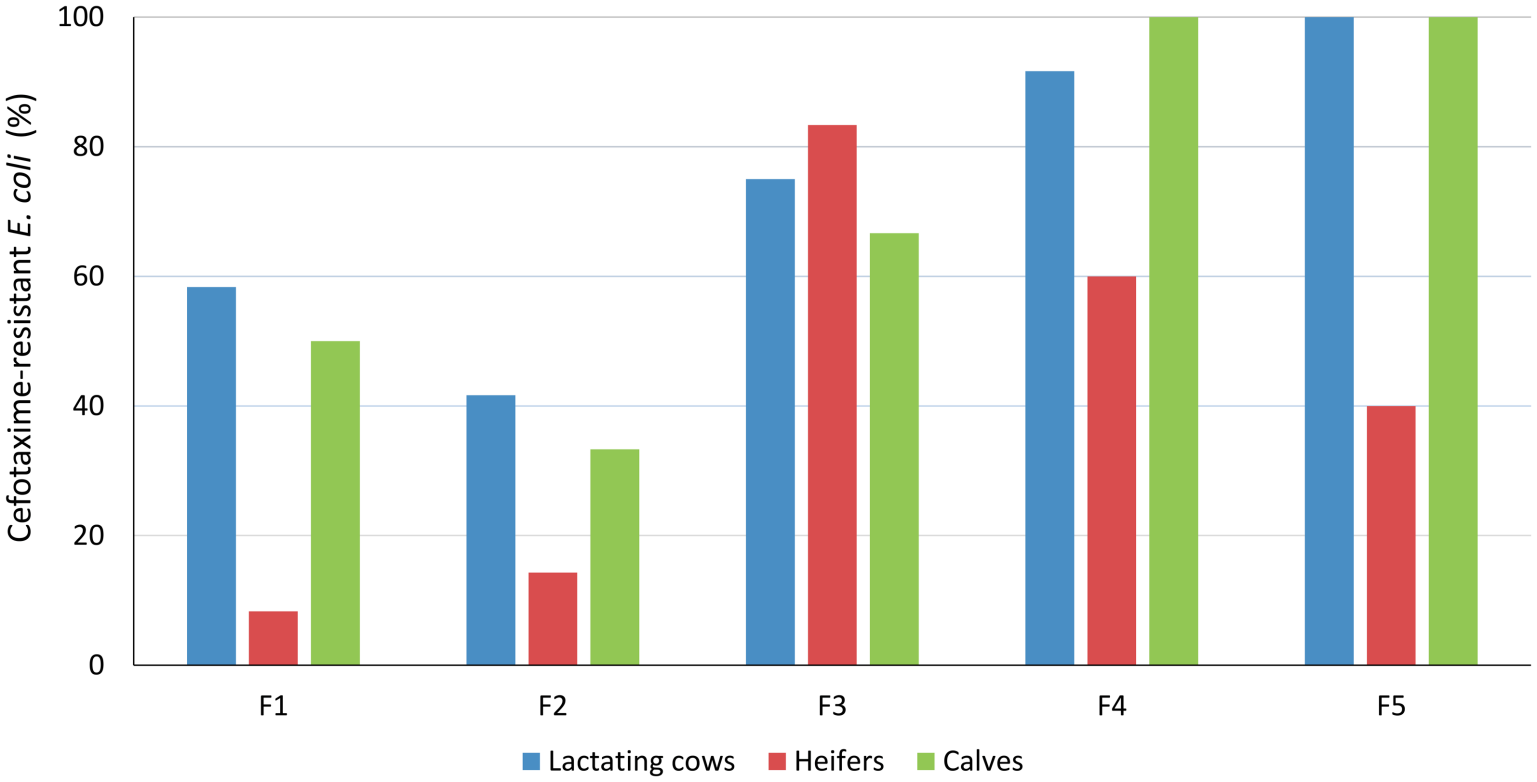
Isolation frequency of cefotaxime-resistant *E. coli* among age groups and farms. Results were based on 12 samplings per farm and age group, except for heifers in F2 and F4 where only 7 and 10 pool samples were collected, respectively, and F5, which dropped out from the study after 5 samplings due to operational changes.

*Escherichia coli* was isolated in cefotaxime-containing media in 92 of the 152 pooled fecal samples analyzed (60.5%) and in the 4 slurry samples collected from F3 and F4. These included samples collected from all farms and age groups, but differences in frequencies among age groups and farms were observed (Figure 1). Overall, isolation frequency of cefotaxime-resistant *E. coli* was higher in lactating cows [OR_adj_ = 4.71 (1.76–12.64), *p* = 0.002] and calves [OR_adj_ = 4.21 (1.59–11.18), *p* = 0.004] compared with heifers, and lower in F1 and F2 compared with the other three farms (LR *χ*^2^ = 21.55, *p* < 0.001).

### The majority of cefotaxime-resistant *Escherichia coli* isolates were also resistant to several other antimicrobials

When available, between 1 and 3 cefotaxime-resistant *E. coli* isolates per age group and sampling date were selected in each farm for antimicrobial susceptibility testing. Thus, 187 isolates from fecal samples (73 isolated from lactating cows, 40 from heifers, and 74 from calves) and 10 isolates from slurry were analyzed. Since isolates had been obtained by selective isolation in a medium containing cefotaxime, they were all resistant to cefotaxime and ampicillin. Most isolates were also resistant to cefepime (99.0%) and ceftazidime (98.0%). Resistance to cefoxitin was detected in 36 isolates (18.3%), but 19 of them displayed a MIC value just one dilution step above the ECOFF. All 197 isolates were susceptible to tigecycline and colistin. Two isolates obtained from the same pool of feces collected from calves in F4 were resistant to all β-lactams tested, including temocillin and carbapenems (ertapenem, imipenem, and meropenem).

Co-resistance to other antimicrobial classes was also observed in most isolates (161/197, 81.7%) and 72.1% (142/197) showed multidrug resistance (MDR, resistance to 3 or more antimicrobial classes). Overall, resistance to tetracycline (53.8%), nalidixic acid (45.7%), ciprofloxacin (66.5%), sulfamethoxazole (69.0%), trimethoprim (48.7%), and chloramphenicol (47.7%) was very frequent, while resistance to gentamicin (29.9%) and azithromycin (14.2%) was lower and mainly associated to F5. The prevalence of resistance to each antimicrobial tested did not differ between age groups. However, statistically significant differences between farms were observed in the occurrence of resistance to several antimicrobials. Compared to other farms, F1 and F5 presented a significantly higher prevalence of tetracycline, chloramphenicol, and trimethoprim (all with *p* < 0.001). Resistance to gentamicin (*p* < 0.001), azithromycin (*p* < 0.001), ciprofloxacin (*p* = 0.002), and nalidixic acid (*p* = 0.009) was higher in F5 than in other farms, while resistance to cefoxitin was significantly higher in F1 and F2 (*p* = 0.003).

### The diversity of phenotypic resistance profiles varied among farms

**Figure 2:**
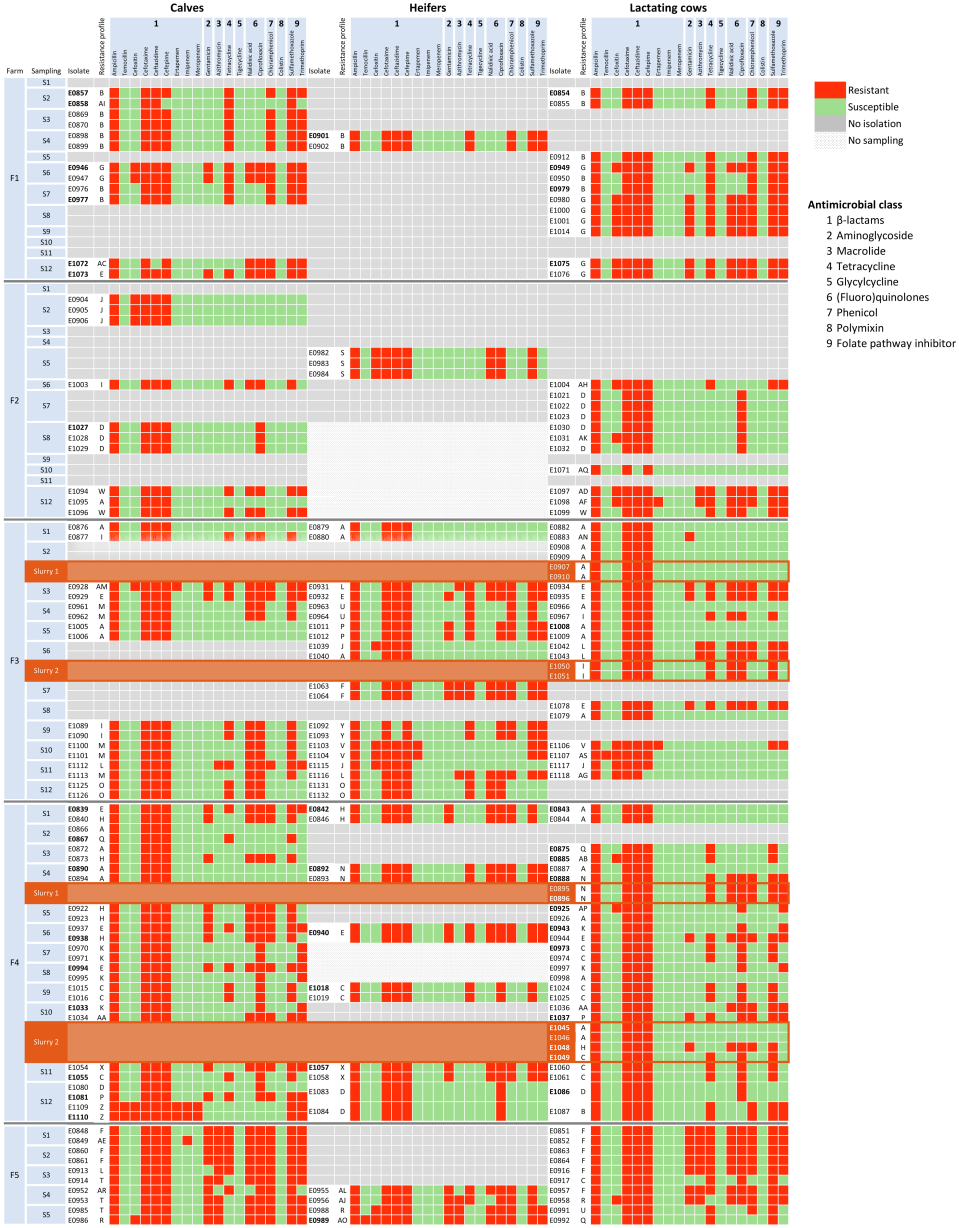
Distribution of AMR phenotypic profiles of the 197 *E. coli* isolates by farm, sampling, and age group. Each phenotypic profile is represented with a letter of the Latin alphabet as described in Supplementary Table S1. Antimicrobial susceptibility, determined by the broth microdilution method and interpreted using epidemiological cutoff values (see text), is shown in green for susceptible, and in red for resistant isolates. Slurry samples are indicated with a different background color (brown) and placed below lactating cows to save space. Antimicrobial classes are indicated with numbers: 1 = β-lactam, 2 = Aminoglycoside, 3 = Macrolide, 4 = Tetracycline, 5 = Glycylcycline, 6 = (Fluoro)quinolone, 7 = Phenicol, 8 = Polymyxin, 9 = Folate pathway inhibitor.

A total of 45 different profiles of microbiological resistance (Supplementary Table S1) resulting from the combination of antimicrobial agents that showed MICs above the ECOFF were observed in the study. Each phenotypic resistance profile was designated a letter of the Latin alphabet, and their distribution within each farm is represented in Figure 2. Within each farm, the number of different profiles ranged between 5 and 16 along the 12 samplings, the lowest diversity being found in F1 (Simpson index = 0.609) and the highest in F4 (Simpson index = 0.905). In F1, resistance to tetracycline, chloramphenicol, sulfamethoxazole, and trimethoprim remained stable during the entire study, whereas resistance to gentamicin, ciprofloxacin, and nalidixic acid was only observed in the second half of the study. This observation might reflect a shift in the circulating resistance profiles, where profile B, which dominated at the beginning of the study in all the age groups, was displaced by profile G in the second half of the study. On the contrary, the highest diversity in resistance profiles was observed in F4, where the three predominant profiles (A, C, and H) coexisted with 12 other profiles, with A and H dominating in the first half of the study, and profile C in the second half. Profile A only included resistance to ESBLs (penicillins and cephalosporins), whereas C and H included resistance to additional antimicrobials (Figure 2; Supplementary Table S1).

In F2, the prevalence of cefotaxime-resistant *E. coli* was the lowest, and fewer isolates were recovered and typed, particularly in heifers. Still, 11 different profiles were identified among 24 isolates, but profile D was the only one recovered in more than one sampling, in S7 in lactating cows and in S8 both in calves and lactating cows. In F3 high diversity in resistance profiles was observed, with a total of 16 different profiles, and a shift in the predominant resistance profiles occurred with time as happened in F4. Finally, the most outstanding feature of isolates recovered in F5 was the MDR pattern of all of them, with resistance to gentamicin and azithromycin being common in all age groups. On the other hand, isolates recovered from slurry samples shared their resistance profiles with isolates from fecal samples collected within the corresponding farms (Figure 2; Supplementary Figure S1).

### Sequences generated by ONT sequencing successfully assembled into complete and circular chromosomes and plasmids

**Figure 3:**
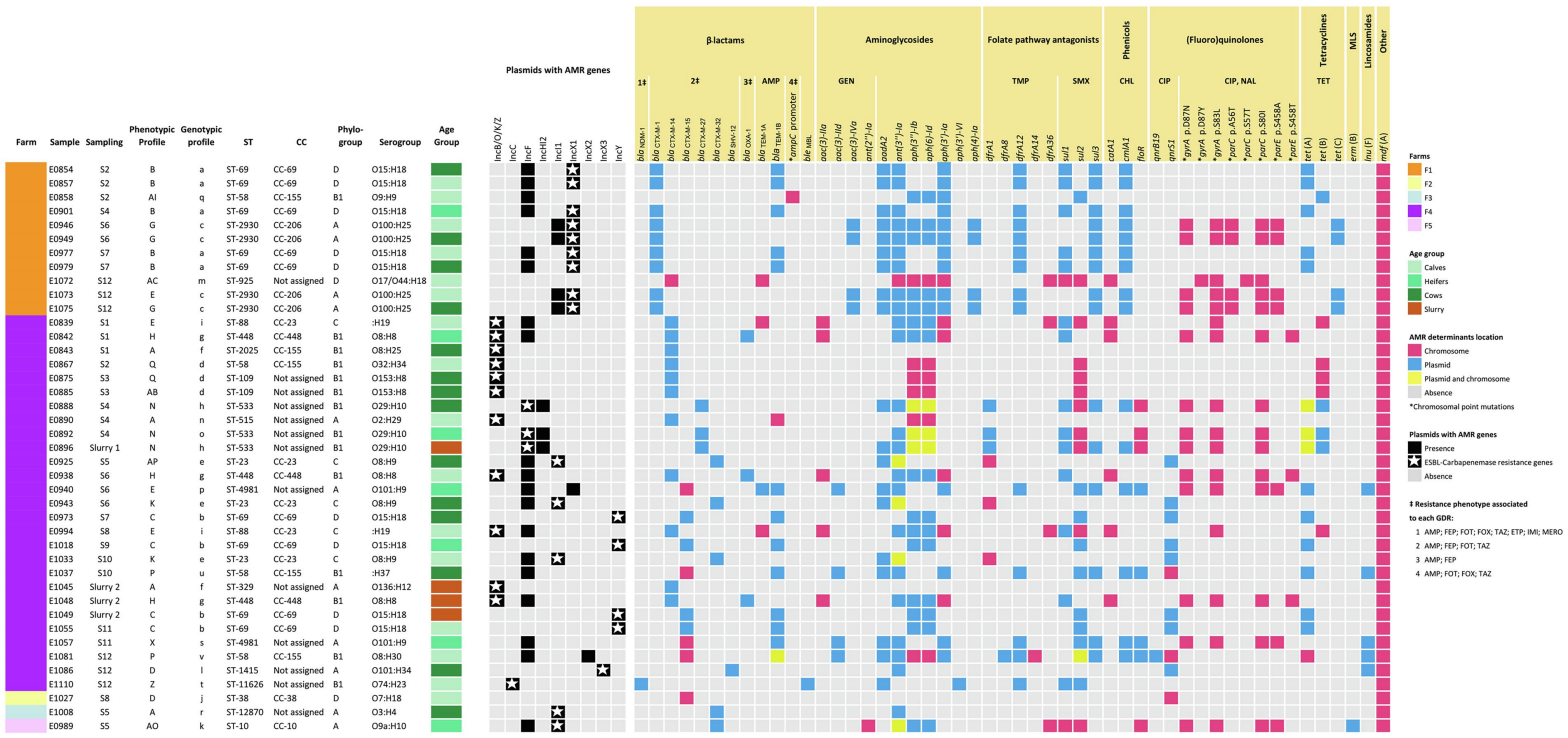
Heatmap showing the distribution of the AMR genes and plasmids detected by WGS (presence or absence and location are indicated as per the legend). Isolates are arranged per farm, sampling, and source (age group or slurry). Additional information including MLST type (ST and CC), phylogroup, and serogroup are included. AMR phenotypic resistance profiles are as indicated in Figure 2 and described in Supplementary Table S1. Each AMR genotypic profile resulting from an identical combination of GDR is represented with a letter of the Latin alphabet in lower case. The resistance phenotypes associated with each GDR are indicated for those antimicrobials tested, which were abbreviated as follows: ampicillin (AMP), cefepime (FEP), cefotaxime (FOT), cefoxitin (FOX), ceftazidime (TAZ), ertapenem (ETP), imipenem (IMI), meropenem (MERO), gentamicin (GEN), trimethoprim (TMP), sulfamethoxazole (SMX), chloramphenicol (CHL), nalidixic acid (NAL), ciprofloxacin (CIP), tetracycline (TET). MLS, macrolide-lincosamide-streptogramin. Descriptions of the phenotypic and genotypic profiles of resistance can be found in Supplementary Tables S1 and S3, respectively.

**Figure 4:**
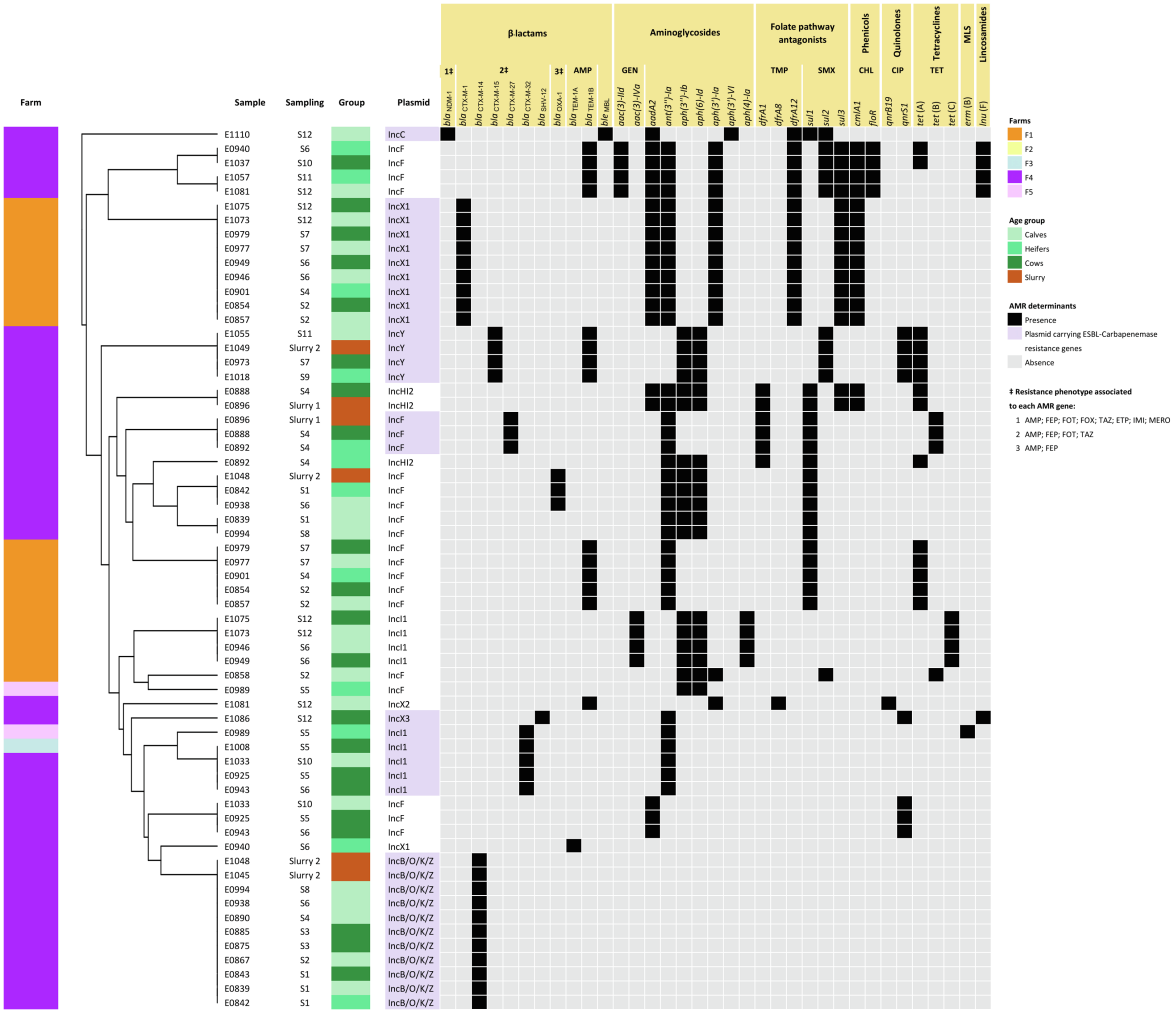
Heatmap showing ARG-harboring plasmids along with a dendrogram illustrating the similarity among plasmids based on their AMR pattern. Plasmids were grouped based on their antimicrobial resistance pattern (ARGs) according to the result of the hierarchical clustering using the average linkage method (UPGMA) on the Jaccard distance matrix. Further information including sampling and source (age group or slurry) is also included.

ONT sequencing provided a median of 60,729 reads per sample (IQR = 22,781–405,021) in a median of 631 Mb per sample (IQR = 501–1,011 Mb) corresponding to a median coverage of 114X (IQR = 84X–182X; Supplementary Table S2). Upon assembly, the 5 isolates sequenced by both Illumina and ONT technologies, and 24 of the 36 ONT sequenced isolates resulted in circularized chromosomes. In all cases, the chromosome size of the assembled draft genome corresponded to the expected size of *E. coli* (median = 4,999,307 bp; IQR = 4,871,651 bp - 5,059,042 bp). Plasmid replicons were identified in a total of 125 contigs that in most cases (120/125, 96.0%) were assembled into complete circular plasmids. At least one plasmid replicon was identified in each isolate. IncF type plasmids were the most common (38/125, 30.4%), followed by IncB/O/K/Z (15/125, 12.0%), IncX1 (13/125, 10.4%), and IncY (13/125, 10.4%) along with 13 other replicon types. Screening for ARGs and SNPs associated with AMR identified 41 acquired ARGs and point mutations (9) in 4 other genes, coding for resistance to antimicrobials representing 9 different classes (Figure 3). The combination of GDRs detected in each isolate resulted in 22 different genotypic profiles of resistance (Supplementary Table S3). Sixty-two plasmids contained at least one ARG (Figure 4). None of the IncL, IncP, IncX4, or Col plasmids carried ARG genes.

### WGS confirmed the predominance of certain genomic subtypes of *Escherichia coli* in F1 and great variability of strains in F4

A selection of isolates, mainly from farms F1 and F4, were analyzed by WGS to confirm whether the distribution of the different AMR profiles within the farms was due to different strains coexisting throughout the sampling period or to successive colonization by different strains. The 41 isolates were assigned to 18 MLST types, including two novel STs, i.e., ST-11626 in F4 and ST-12870 in F3. In F1, the 11 isolates tested belonged to 4 ST types, and 2 of them (ST-69 and ST-2930) included more than one isolate (Figure 3). Thus, ST-69 was represented by 5 isolates from the 3 age groups recovered at samplings S2, S4, and S7, which were identical in all other features, i.e., phylogroup (D), serotype (O15:H18), phenotypic resistance profile (B), and genotypic profile (a). ST-2930 included 4 isolates recovered in samplings S6 and S12 from calves and lactating cows that also shared all their genetic features, i.e., they were all assigned to phylogroup A, serotype O100:H25, and genotypic profile c. However, they split into two phenotypic resistance profiles differing only in susceptibility to FOX (profile E, 1 susceptible isolate, MIC_FOX_ = 2; and profile G, 3 resistant isolates, MIC_FOX_ = 16). The remaining two isolates sequenced (E0858 and E1072) were recovered during the second and last samplings, and had unique features.

In F4, 27 isolates were sequenced and assigned to 4 phylogroups (A, B1, C, and D) and 13 different ST types. Eight of the ST types were identified in more than one isolate (*n* = 2–4) and 5 were represented by a single isolate (Figure 3). As in F1, the most prevalent MLST type was ST-69. It included isolates recovered from lactating cows (S7), heifers (S9), calves (S11), and slurry. They all shared the same phylogroup (D), serotype (O15:H18), and genotypic (b) and phenotypic AMR profiles (C), suggesting that the same clone spread after sampling 7 in all animal groups and was also detected in slurry. As described in F1, differences associated with FOX among otherwise similar isolates were also observed in F4 within ST-23 (profiles AP and K) and ST-109 (profiles AB and Q). Other identical clones infecting several animals in F4 were those with MLST types ST-88 (*n* = 2), and ST-448 (*n* = 3).

On the other hand, several isolates with identical ST differed in other features (Figure 3). These included differences in resistance due to the carriage of ARG-harboring plasmids (i.e., ST-4981 and ST-69) and occasionally also in chromosomally encoded features (i.e., ST-58 isolates, which differed in ARG-harboring plasmids as well as serotype and chromosomally encoded ARGs). Finally, the isolates from F2, F3, and F5 were unique in all their features. When virulence genes were examined, patterns of presence/absence were highly conserved within ST types, with ST-58 and ST-69 being the only exceptions (Supplementary Table S4). Thus, for these epidemiologically related isolates, the typing scheme based on the presence/absence of virulence genes confirmed the diverse genetic profile inferred from the combination of all other features (phylogroup, serotype, and GDR profile).

### Resistance to cephalosporins was mainly due to plasmid-encoded *bla*_CTX-M_ genes. F1 differs from F4 regarding the diversity and location of cephalosporin resistance genes

ARG-harboring plasmids were present in all but two of the isolates (E1072 and E1027); 16 isolates carried a single plasmid and 23 carried 2 types of plasmids with ARGs. Overall, ARGs were present in 9 different types of plasmids, and 7 of them harbored ESBL-encoding genes, alone or in combination with several other ARGs (Figure 4). GDRs associated with ESBL production were only sporadically located in the chromosome. These included *bla*_CTX-M-14_ (*n* = 1), *bla*_CTX-M-15_ (*n* = 5), and the point mutation (nt 42 C → T) in the *ampC* promoter (*n* = 1). However, differences between farms were found concerning the diversity and location of these genes.

In F1, the most prevalent ESBL-encoding gene was *bla*_CTX-M-1_ (9/11 isolates), present in isolates recovered from all animal groups and at different sampling times along the study (Figure 3). This gene was located in IncX1 type plasmids which always carried the same repertoire of ARGs. These plasmids were structurally compared using MAUVE and showed a high degree of similarity demonstrating the presence of largely conserved collinear coding blocks (data not shown). Hence, in addition to *bla*_CTX-M-1_, IncX1 plasmids harbored the aminoglycoside resistance genes *aadA2*, *ant(3″)-Ia*, and *aph(3′)-Ia*, a trimethoprim resistance gene (*dfrA12*), a sulfamethoxazole resistance gene (*sul3*), and a chloramphenicol resistance gene (*cmlA1*; Figure 4). This plasmid was present in all isolates assigned to ST-69 and ST-2930.

In F4, a higher diversity of ESBL-encoding genes was observed (Figure 3). These included *bla*_CTX-M-14_ (*n* = 11), *bla*_CTX-M-15_ (*n* = 8), *bla*_CTX-M-27_ (*n* = 3), *bla*_CTX-M-32_ (*n* = 3), and *bla*_SHV-12_ (*n* = 1). In addition, *bla*_NDM-1_ was detected in one isolate (E1110). All were located in plasmids except for 4 chromosomally encoded *bla*_CTX-M-15_. The *bla*_CTX-M-14_ gene was always located in IncB/O/K/Z type plasmids that did not carry any additional ARGs (Figure 4). This plasmid was found in *E. coli* of different ST, genotypic and phenotypic profiles, isolated from slurry and animals of all age groups throughout the study. The *bla*_CTX-M-15_ gene, which was also detected in all animal groups and environmental samples, was the predominant ESBL-encoding gene in isolates recovered in the second half of the study. This gene was located in IncY plasmids (all 4 ST-69 isolates) or in the chromosome (ST-4981 and ST-58). Besides *bla*_CTX-M-15_, IncY plasmids harbored 6 other ARGs (Figure 4). The *bla*_CTX-M-27_ gene was detected in the IncF plasmid of 3 ST-533 isolates, along with 5 other identical ARGs. Three isolates (ST-23) carried the *bla*_CTX-M-32_ gene in an Incl1 plasmid, and the *bla*_SHV_ gene was present in an IncX3 plasmid in one isolate recovered from lactating cows in the last sampling. The isolation in F4 of a carbapenem-resistant *E. coli* harboring the *bla*_NDM-1_ gene in an IncC plasmid was a significant finding extensively reported elsewhere (Tello et al., 2022).

### Cephalosporin-resistant *Escherichia coli* isolates carried additional plasmids with ARGs and exhibited other chromosomally encoded GDRs

In F1, ST-2930 isolates, besides IncX1, also carried an IncI1 plasmid that harbored another 5 ARGs [*aac(3)-IVa*, *aph(3″)-Ib*, *aph(6)-Id*, *aph(4)-Ia*, and *tet*(C)], while ST-69 isolates carried an IncF plasmid that harbored another four different ARGs [*bla*_TEM-1B_, *ant(3″)-Ia*, *sul1* and *tet*(A)]. A different IncF plasmid with a different collection of ARGs was present in one isolate (E0858). This isolate also carried a chromosomally encoded mutation in the *ampC* promoter. No plasmids were detected in the remaining isolate of F1 sequenced (ST-925), which harbored several GDRs in its chromosome, including *bla*_CTX-M-14_ (Figure 3). Resistance to (fluoro)quinolones was always associated with point mutations in the gyrase and topoisomerase genes (*gyrA*, *parC*, and *parE*) and only observed in *E. coli* strains assigned to ST-2930 and ST-925 (Figure 3).

In F4, the 3 ST-23 isolates, which carried an Incl1 plasmid harboring the *bla*_CTX-M-32_ gene, also carried an IncF plasmid, resulting in an identical genotypic profile. Instead, ST-533 isolates (E0888, E0892, and E0896) carried a second plasmid (IncHI2) with a different repertoire of ARGs (Figure 4). These IncHI2 plasmids were structurally compared (Supplementary Figure S2) and showed extensive sequence similarity, but E0896 lacked an 11.000 bp fragment that included *aadA2*, *cmlA1*, *ant(3″)-Ia*, and *sul3* genes which was present in E0888 and E0892. Other genes coding only for resistance to narrow-spectrum β-lactamases like *bla*_OXA-1_ (*n* = 3), *bla*_TEM-1A_ (*n* = 4), and *bla*_TEM-1B_ (*n* = 14) were mostly located in IncF plasmids, and less frequently in the chromosome or other type of plasmids such as IncX2 and IncY (Figures 3, 4). Resistance to (fluoro)quinolones in F4 was associated with point mutations in the gyrase and topoisomerase genes (*gyrA*, *parC*, and *parE*) in 10 isolates, and with the *qnrS1* gene in another 10 (along with *qnrB19* in one of them). Interestingly, the gene that codes for resistance to lincosamides, *lnuF*, was present in 5 isolates recovered from all age groups in the second half of the study. *lnuF* was always located in IncF and IncX3 plasmids (Figure 4).

## Discussion

This longitudinal study was designed to monitor the occurrence of ESBL-/AmpC-/CP-producing *E. coli* and their antimicrobial resistance profiles in apparently healthy animals in dairy cattle farms for over 16 months. Longitudinal surveillance allows the assessment of the bacterial population dynamics throughout time, enabling the detection of emerging genotypes and changes in the AMR profiles over time. The longitudinal survey presented here encompassed five farms that represented the style of farming in the Basque Country, and therefore, might provide a useful understanding of the regional situation regarding cephalosporin-resistant *E. coli* distributions and AMR transmission dynamics.

In a previous cross-sectional survey conducted in the Basque Country in 2014–2016, ESBL/AmpC producers were isolated in 32.9% of the 82 dairy cattle herds tested (Tello et al., 2020). Here, cephalosporin-resistant *E. coli* were detected in all the five investigated dairy cattle farms, surely due to the more intensive longitudinal sampling strategy used that comprised 12 samplings and three age groups. Isolation frequency varied along time, as well as among farms and age groups. Both calves and lactating cows had a higher prevalence of cephalosporin-resistant *E. coli* than heifers, but no difference was observed between them. This could be associated with age-related differences in management practices. Pregnant heifers and dry cows had access to the outside pastures, whereas lactating cows were permanently housed indoors, where increased infection pressure and a higher probability of recirculation of resistant isolates occur. In this sense, ruminants raised under less intensive management systems have been associated with a lower prevalence of infection with cefotaxime-resistant *E. coli*, e.g., beef cattle and sheep in the Basque Country (Tello et al., 2020) and elsewhere (Hille et al., 2017; Collis et al., 2019). The higher incidence found in lactating cows compared with heifers could also be explained by the continuous and prolonged exposure of older cows to antimicrobials used to treat intramammary and other infections during their lifespan. These treatments include the commonly used cephalosporins, which do not require a withdrawal period for milk. On the other hand, calf management practices differed from those in heifers and lactating cows. Calves are kept in different housing facilities and are administered a different diet. Moreover, calves are susceptible to different diseases such as neonatal diarrhea and pneumonia, which are the main reasons for antimicrobial treatment in this age group. Besides, young calves rapidly acquire antibiotic-resistant *E. coli*, which are often multiresistant (Hordijk et al., 2013; Gay et al., 2019), and their resistome has been reported to be more diverse than that of adult cattle (Noyes et al., 2016).

Antimicrobial use (AMU) in food animals has been linked to an increased prevalence of resistant bacteria, but this relation depends on the antimicrobial class, microorganism, and sector (ECDC, EFSA, and EMA, 2021). Here, in the absence of detailed records of AMU, differences extracted from the questionnaires were related to mastitis treatments and DCT. Fluoroquinolones were the antibiotics of choice for mastitis treatment in F1, F3, and F5, the combination of parenteral enrofloxacin with an intramammary ointment containing cefquinome was common practice in F4, and no antimicrobials were used to treat mastitis in F2. On the other hand, the antimicrobials used for blanket DCT to control mastitis were penicillins and aminoglycosides in F1 and F2, and a first-generation cephalosporin in F3, F4, and F5. This could somehow explain the higher prevalence of cephalosporin-resistant *E. coli* found in F3, F4, and F5 compared to F1 and F2. Differences in farm infrastructure and management practices (e.g., vaccine programs and hygiene) may impact animal disease incidence and, consequently, influence the use of antimicrobials and the subsequent increase in AMR prevalence.

Antimicrobial susceptibility testing of 197 cefotaxime-resistant *E. coli* isolates identified 72.1% of them as MDR. This is not unexpected since ESBL-producing *E. coli* are commonly co-resistant to other classes of antimicrobials (Seiffert et al., 2013). However, the diversity of phenotypic resistance profiles varied among farms. Therefore, to thoroughly compare the relationship of the circulating strains, a selection of isolates from the two farms that showed the lowest (F1) and largest (F4) AMR profile diversity were further whole-genome characterized. This analysis identified certain isolates with phenotypic AMR profiles that differed only in their susceptibility to FOX; the FOX-resistant isolates showed a MIC value of just a single two-fold dilution above the ECOFF (MIC_FOX_ = 16 mg/l), and, therefore, within the widely accepted margin of error of the microdilution method. These isolates did not carry any GDR associated with AmpC production, and based on WGS results (ST, phylogroup, serotype, GDR, and virulence genes) these isolates could be considered the same strains as their FOX-susceptible counterparts within the same ST type. The opposite situation, i.e., isolates with the same phenotypic profile but clearly different ARGs was also observed. This occurred in F4 and was due to changes in the chromosome and the carriage of different plasmids (ST-58) or the loss of a fragment within an otherwise similar plasmid (ST-533). Isolates with different ST and serotypes that shared the same ARGs were also found.

Even though the genomic data provided in this study represents only two farms, *bla*_CTX-M-1_, *bla*_CTX-M-14_, and *bla*_CTX-M-15_ were the most common ESBL-encoding genes, as reported in a previous cross-sectional study carried out in the Basque Country (Tello et al., 2020). Noteworthy was the detection of a gene coding for CP production in F4. The identification, in the frame of this study, of a *bla*_NDM-1_-carrying *E. coli* was described in more detail elsewhere (Tello et al., 2022). Previous to this study, CP-producing *E. coli* had not been detected in food-producing animals in the Basque Country, and *bla*_NDM-1_-carrying *E. coli* had never been isolated from cattle neither in the Basque Country nor elsewhere.

ESBL-/CP-encoding genes were mostly located in plasmids, with an apparent association of each gene with certain types of plasmid. IncB/O/K/Z plasmids are frequently found in *E. coli* from animal sources and have been associated with the spread of *bla*_CTX-M-14_ in Europe, especially in Spain and the UK (Rozwandowicz et al., 2018). IncF is the most frequently described plasmid type from human and animal sources (Rozwandowicz et al., 2018) and encodes different *bla*_CTX-M_ variants. Here, IncF was the most abundant plasmid but only sporadically carried ARG coding for resistance to ESBL, specifically *bla*_CTX-M-27_ gene, an association already described in cattle (Tadesse et al., 2018). Other ESBL-encoding gene and plasmid associations found here, such as *bla*_CTX-M-1_ in IncX1, *bla*_CTX-M-15_ in IncY, *bla*_CTX-M-32_ in Incl1, and *bla*_SHV_ in IncX3 plasmids, are not so frequently described (Rozwandowicz et al., 2018). In the cross-sectional study previously carried out in the region (Tello et al., 2020), most of the ESBL/AmpC gene-carrying plasmids were identified as IncI1, but since Illumina was the sequencing technology then used, the type of many of the plasmids could not be assigned. Here, using long-read ONT sequencing, most of the genomes (both the chromosomes and plasmids) were completely sequenced and circularized, allowing a better characterization of plasmids, which is one of the main advantages of this technique (Wick et al., 2017a).

Genome sequencing and characterization of this selection of isolates allowed elucidation of whether transmission of ESBL genes was the result of the persistence of certain strains or multiple source contamination. In F1, only four different strains were identified, two of them being recovered multiple times and from all age groups. One predominated during the first half of the study and was then replaced by a very different strain. Their chromosomally encoded features (all 7 ST alleles, CC, phylogroup, serotype, virulence genes profile, and point mutations associated with quinolone resistance) were completely different, but both carried the same ESBL-encoding gene (*bla*_CTX-M-1_) harbored by an identical IncX1 plasmid. Further differences between both strains were due to genes present in different additional plasmids. These results may reflect an endemic situation where, due to clonal expansion, just a few strains persisted in the farm over a long time thus giving the opportunity for plasmid transfer. Conversely, the situation in F4 was completely different. Although a few genotypes persisted for some time, there was a large diversity of genotypes carrying multiple and diverse GDRs both in the chromosome and in different plasmids, likely due to multiple source contamination events. Yet, different *E. coli* isolates containing the same type of plasmids that carry the same repertoire of ARGs were also identified (e.g., IncB/O/K/Z in 7 different STs). This strongly suggested that horizontal transfer of ESBL-carrying plasmids occurred within the farm.

## Conclusion

In conclusion, this study illustrates the within-farm diversity and dynamics of cefotaxime-resistant *E. coli* over time in dairy cattle, and shows the power of genomic surveillance in deciphering the complex epidemiology underlying multidrug resistance dissemination within a farm. Despite the differences observed between both farms, the presence of certain plasmid types with the same repertoire of ARGs in different *E. coli* STs might be indicative of the occurrence of horizontal transfer of such plasmids among strains circulating within the farms. AMU, environmental selection pressure, or co-selection with other advantageous genes might drive these events. Although we cannot rule out the existence of certain niche-specific clones that are better adapted to the calf intestinal environment, we found that the more widespread clones could readily infect animals of all age groups. Recommendations for the implementation of biosecurity measures to prevent the introduction of ESBL-producing *E. coli* and management protocols that limit contact between animals of different age groups were made to farmers to avoid cross-contamination and the spread of resistant bacteria. Considering the public health importance of ESBL-producing *E. coli* both as pathogens and as vectors for resistance mechanisms, the presence of β-lactamase- and other AMR-encoding genes in plasmids that can be readily transferred between bacteria is a concern that highlights the need for One Health surveillance.

## Data availability statement

The raw sequencing data presented in this study can be found online at the NCBI Sequence Read Archive (SRA) database, associated with the BioProjects PRJNA833969 and PRJNA680938. The accession number(s) can be found in Table S5 in the Supplementary material.

## Ethics statement

Ethical review and approval by the Ethics Committee for Animal Experimentation was not required for the animal study because sample collection was carried out by veterinary practitioners strictly following Spanish ethical guidelines and animal welfare regulations (Real Decreto 53/2013) as part of their routine veterinary practice. Informed consent was obtained from the farm owners at the time of sample collection. Written informed consent was obtained from the owners for the participation of their animals in this study.

## Author contributions

AH conceived and coordinated the study. BO and MT performed laboratory analyses. JL, MO, and MT carried out bioinformatic data analyses. MO performed statistical analyses. AH, MO, and MT interpreted the data and wrote the manuscript. BO and JL contributed to the manuscript revision. All authors contributed to the article and approved the submitted version.

## Funding

This work was supported by the Basque Government: The Department of Economic Development, Sustainability, and Environment (URAGAN 17-00892). MT is the recipient of a predoctoral fellowship from the Basque Government (Department of Economic Development, Sustainability, and Environment).

## Conflict of interest

The authors declare that the research was conducted in the absence of any commercial or financial relationships that could be construed as a potential conflict of interest.

## Publisher’s note

All claims expressed in this article are solely those of the authors and do not necessarily represent those of their affiliated organizations, or those of the publisher, the editors and the reviewers. Any product that may be evaluated in this article, or claim that may be made by its manufacturer, is not guaranteed or endorsed by the publisher.

## Footnotes

^1^ https://eur-lex.europa.eu/legal-content/EN/TXT/PDF/?uri_CELEX:32013D0652&from_EN

^2^ http://www.snapgene.com/

^3^ https://www.geneious.com
