# Supplementary material for: Within-farm dynamics of ESBL-producing Escherichia coli in dairy cattle: Resistance profiles and molecular characterization by long-read whole-genome sequencing
Source: Front Microbiol. 2022 Jul 28;13:936843. doi: 10.3389/fmicb.2022.936843 (PMC9366117; doi:10.3389/fmicb.2022.936843)
Supplement: Supplementary file 1 [file Table_1.DOCX]

Supplementary Material

**Supplementary Figure S1.** Venn diagrams showing the distribution of AMR phenotypic profiles for animal groups on each farm. AMR phenotypic profiles which are also detected in slurry samples are marked with an asterisk.


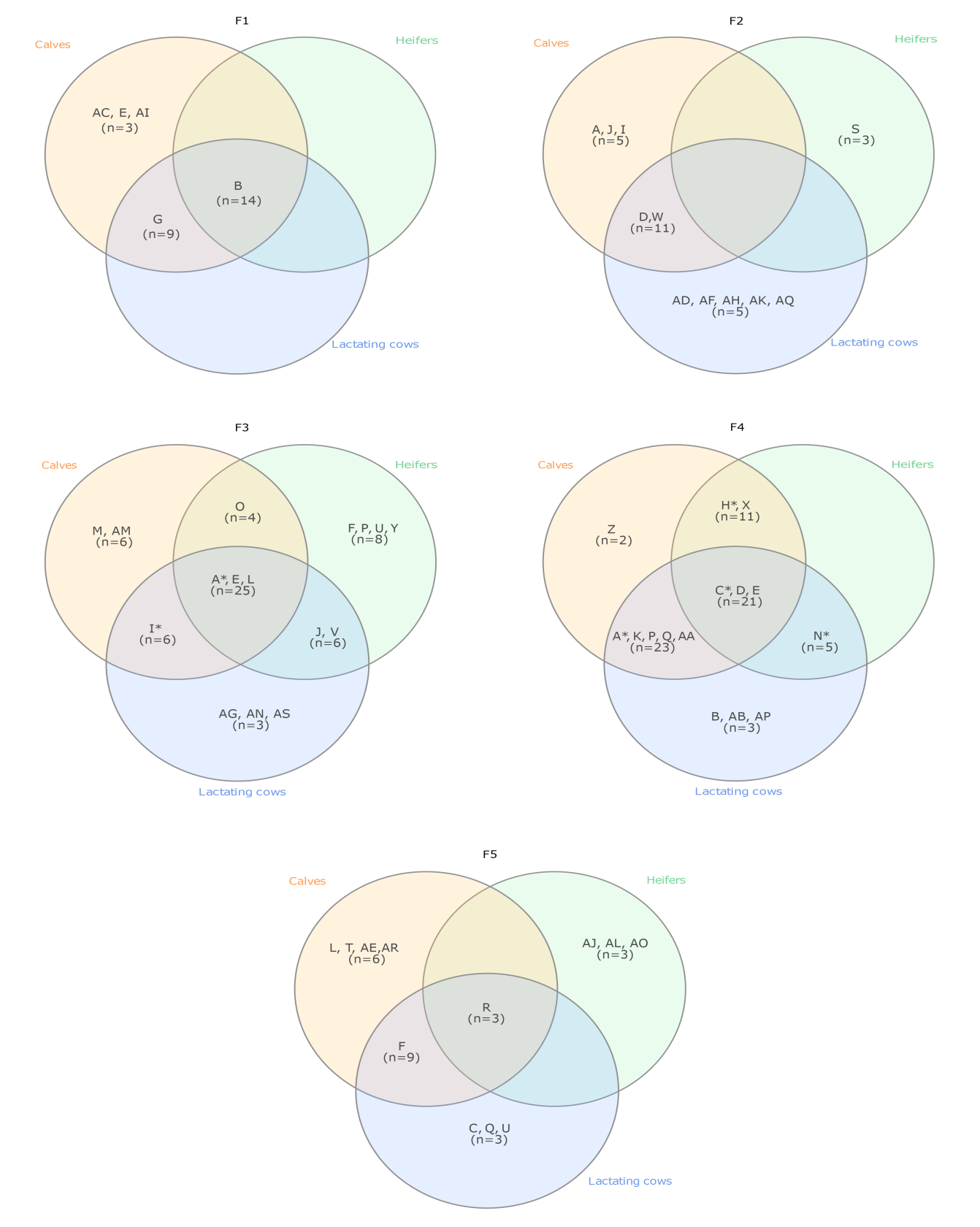


**Supplementary Figure S2.** Structural comparison of IncHI2 plasmid in isolates E0888, E0892, and E0896. (a) Circular comparison of plasmids using Blast Ring Image Generator (BRIG), using E0896 IncHI2 plasmid as a reference. The location of ARGs is indicated with an arrow. (b) Comparison of ARG region in IncHI2 plasmids of E0888 and E0892. Coding sequences (CDS), represented by arrows indicating the translational direction, are named above and colored as follows: blue, insertion sequences (IS); green, AMR genes; brown, genes with other functions; and grey, hypothetical proteins. IS designations are followed by the family name in brackets. Annotations were graphically depicted using SnapGene (v.5.2.4) (<http://www.snapgene.com/>).

**
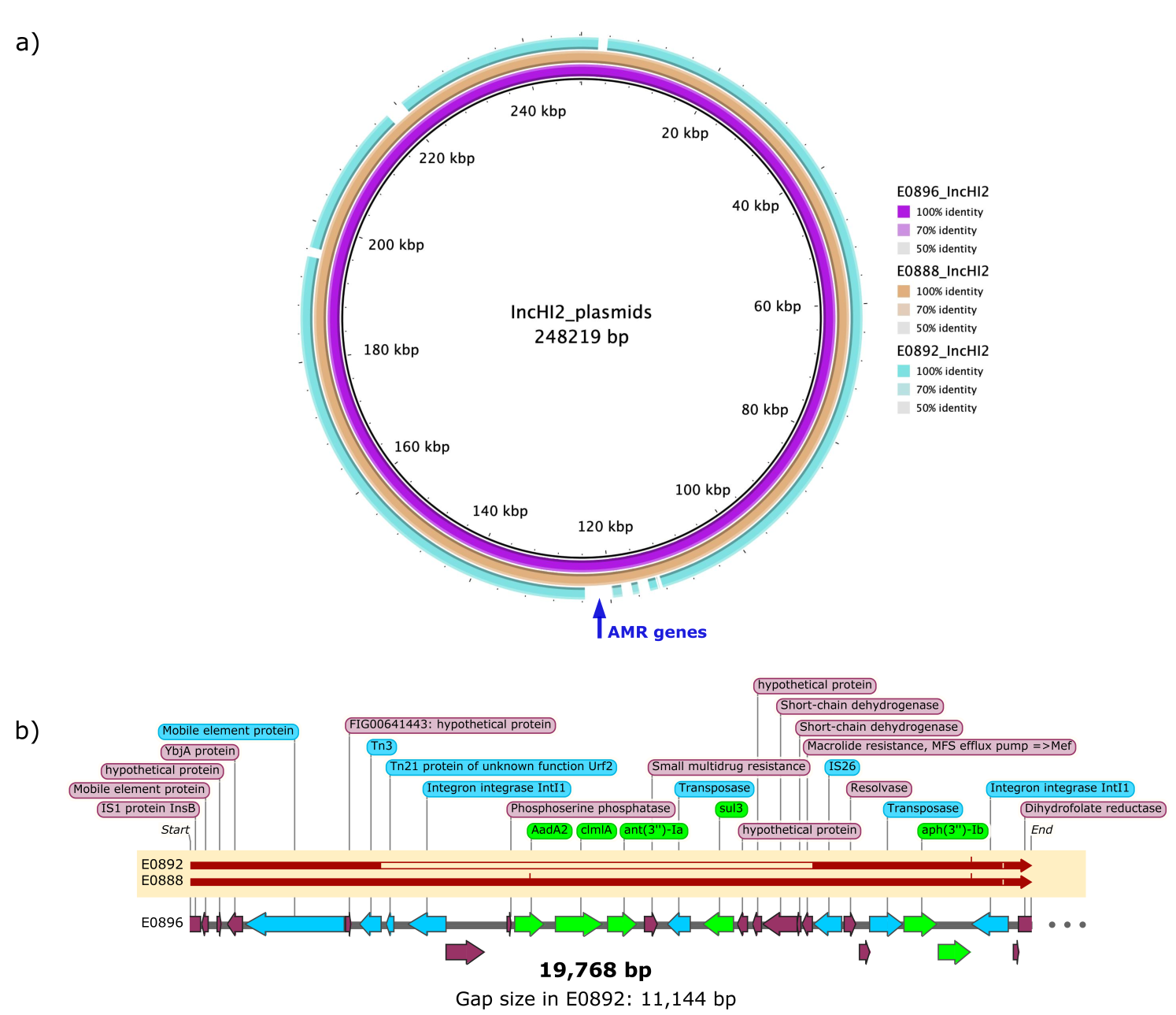
**

# Supplementary Data

The supplementary data file includes the following tables:

**Table S1.** List and abundance of the different AMR phenotypic profiles observed.

**Table S2.** Overview of WGS read output data and assembly for each sample.

**Table S3.** List and abundance of the different AMR genotypic profiles observed.

**Table S4.** Heatmap showing the distribution of virulence factors detected by WGS in each isolate.

**Table S5.** List of NCBI accession numbers associated to uploaded raw data.
